# Parental Factors Associated With Internet Gaming Disorder Among First-Year High School Students: Longitudinal Study

**DOI:** 10.2196/33806

**Published:** 2022-11-08

**Authors:** Rui She, Youmin Zhang, Xue Yang

**Affiliations:** 1 Department of Rehabilitation Sciences The Hong Kong Polytechnic University Hong Kong China; 2 The Jockey Club School of Public Health and Primary Care Faculty of Medicine The Chinese University of Hong Kong Hong Kong China

**Keywords:** internet gaming disorder, adolescents, parental factors, longitudinal study, parenting, gaming, gaming disorder, health intervention, treatment, mental health

## Abstract

**Background:**

Parents play central roles in adolescents’ socialization, behavioral development, and health, including the development of internet gaming disorder (IGD). However, longitudinal research on the parental predictors of adolescent IGD is limited.

**Objective:**

This study aimed to investigate the reciprocal associations between various parental factors and adolescent IGD using 2-wave cross-lagged models.

**Methods:**

A sample of 1200 year-one high school students in central China completed a baseline assessment in 2018 (mean age 15.6 years; 633/1200, 52.8% male) and a follow-up survey in 2019. IGD was measured using the 9-item DSM-5 IGD Symptoms checklist. Perceptions related to parental variables, including psychological control, parental abuse, parental support, and the parent-child relationship, were also collected from the adolescents.

**Results:**

Of all the participants, 12.4% (148/1200) and 11.7% (140/1200) were classified as having IGD at baseline (T1) and follow-up (T2), respectively. All 4 cross-lagged models fit the data well (range for the comparative fit index .91-.95; range for the standardized root mean square residual .05-.06). Parental support (*β*=–.06, *P*=.02) and parental abuse (*β*=.08, *P*=.002) at T1 predicted IGD symptoms at T2, while parental psychological control (*β*=.03, *P*=.25) and a positive relationship with parents (*β*=–.05, *P*=.07) at T1 had nonsignificant effects on IGD symptoms at T2, when controlling for background variables. In addition, IGD symptoms at T1 did not predict parental factors at T2.

**Conclusions:**

The findings suggest that parental factors may be significant predictors of adolescent IGD. Health interventions should consider involving parents to increase the effectiveness of treatment to prevent and reduce adolescent IGD.

## Introduction

### Background

Problematic internet gaming has become increasingly common with the development of technological progress. Internet gaming disorder (IGD) has been identified in the Diagnostic and Statistical Manual, Fifth Edition (DSM-5) as a condition warranting further research before including it as a formal disorder [1]. In the 11th Edition of the International Classification of Diseases and Related Health Problems (ICD-11) released in February 2022, gaming disorder (both online and offline) is defined as a disorder characterized by 3 criteria: impaired control over gaming, increased priority given to gaming, and continuation or escalation of gaming despite the occurrence of negative consequences for at least 12 months [2]. Compared with adults, adolescents are especially vulnerable to IGD because of their immature cognitive control and lack of proper coping techniques [3]. Adolescence is a period full of stressful challenges involving extensive physical, psychosocial, and interpersonal changes, which might also predispose individuals to the risk of IGD [4]. Such challenges could be intensified for first-year students entering high school, a time when they also have less academic stress and more time on the internet [5]. First-year high school students have reported a higher prevalence of IGD than their counterparts in higher grades [6]. IGD has been widely reported to affect adolescents’ mental and physical health and lead to negative consequences, such as academic failure, poor sleep quality, social anxiety, and sedentary lifestyles [7-9]. As the adverse effects of addictive behavior (eg, IGD) may continue throughout adulthood, it is essential to clarify the determinants of adolescent IGD to inform effective prevention and intervention programs.

Family and parents play central roles in adolescents’ socialization, behavioral development, and health. Parents behave as models for their children, engage in activities with them, monitor their children's behaviors, and provide support and encouragement that can result in behavioral change and positive health outcomes [10]. Notably, the Chinese culture is influenced by the traditional Confucian philosophy, which emphasizes children’s full respect and obedience toward their parents, leading to more parental psychological control practices in the Chinese culture. Thus, parental factors could be especially prominent in the development of IGD among Chinese adolescents, such as parental modeling of online gaming, parental control, family relationships, and functioning [11]. Additionally, many child development theories (eg, transactional model and social coercion theory) suggest that parent-child influences may be bidirectional, as child development reflects continuous dynamic interactions of the child and his or her social settings, including their interactions with parents [12,13]. Therefore, adolescents’ internet gaming behavior may also affect parents’ responses, parents’ behavior, and their relationship with their parents. However, previous studies have largely comprised cross-sectional designs and rarely examined the bidirectional associations between parental factors and adolescent IGD. In this longitudinal study, we examined the reciprocal relationships between 4 parental factors (ie, parental psychological control, parental abuse, parental support, and parent-child relationship) and adolescent IGD.

### Parental Factors and IGD

A number of empirical studies have suggested that parental psychological control is associated with an increased risk of IGD [11,14-16]. Parental psychological control refers to the use of pressure to control children’s behavior by overly regulating their thoughts or feelings, which interferes with their ability to develop identity and autonomy [14]. According to self-determination theory, humans have basic psychological needs for competence, autonomy, and relatedness that are essential for optimal development and functioning [17]. Parental psychological control may intrude upon children’s basic psychological needs, especially for autonomy, which could result in a tendency toward excessive use of online games to satisfy those needs [18]. In addition to psychological control, some studies have indicated that parental abuse may be more prevalent in Chinese versus Western families, which was associated with more adolescent risky behaviors [19,20]. We only identified 1 cross-sectional study reporting that parental physical and verbal abuse was positively associated with IGD [11].

In contrast, parental support and good parent-adolescent relationships are protective factors against adolescent IGD [11,14,15], as they reduce stress, facilitate adaptive coping, and prevent adolescents from unhealthy behaviors [21]. Recent studies indicated that individuals with IGD evaluated their family functioning and cohesion more negatively than nonaddicted individuals [22]. For instance, 3 longitudinal studies showed that better parent-child relationships and social support could protect adolescents from developing problematic gaming behaviors [23-25], although 1 study with university students in Macao reported that social support including family support did not predict IGD at follow-up [26].

### Bidirectional Associations Between Parental Factors and IGD

It is also likely that adolescent behavior problems (eg, IGD) can arouse family conflict and lead to decreased parental support [27]. Parents might practice less monitoring when they feel that their adolescents can control their online activities, which implies that the causality between parental control and IGD might go in both directions [28]. However, longitudinal data on how adolescent IGD contributes to parent-child interactions are inadequate. A few studies have investigated bidirectional associations between parental factors and IGD, suggesting that IGD symptoms elicit ineffective or negative parental responses, which may further exacerbate problematic gaming. Two longitudinal studies demonstrated a reciprocal association between parental behavior or psychological control and IGD among Chinese adolescents [18,23], whereas other studies reported unidirectional associations between adolescent IGD and parental anxiety and between parental attachment and adolescent IGD instead of bidirectional associations [29,30]. Given the mixed findings in the literature, more longitudinal studies to disentangle the relationship between various parental factors and adolescent IGD are greatly warranted to fill the knowledge gap.

### This Study

In this longitudinal study, we aimed to investigate the relationships between a wide range of parental variables (ie, parental psychological control, parental support, parent-child relationship, and verbal or physical abuse by parents) and IGD among Chinese adolescents in their first year of high school. It was hypothesized that (1) a positive relationship with parents and parental support would negatively predict adolescent IGD; (2) parental psychological control and parental abuse would positively predict adolescent IGD; and (3) IGD would predict worse parental responses to children, including a worse parent-child relationship, poorer support from parents, and more parental abuse and parental psychological control.

## Methods

### Study Design and Participants

This study included a convenience sample recruited from 4 high schools in Kaifeng, Henan province in China. The inclusion criteria were (1) first-year high school students, (2) willing to participate in the baseline and follow-up studies, and (3) Chinese speakers. The baseline survey was conducted during the first year of high school for participants in 2018 (T1), while the 1-year follow-up survey (T2) was conducted in the second year of high school. In total, 1239 adolescents completed the baseline survey, and 1200 adolescents completed the follow-up survey (follow-up rate 96.7%); 1200 individuals with complete baseline and follow-up data were included in the final analysis.

### Procedures

Participants completed a self-reported questionnaire that took about 15 minutes to complete in the classroom setting. The survey was facilitated by research assistants but in the absence of teachers. Participation was voluntary, and we guaranteed that there were no penalties for refusals. The confidentiality of the data was guaranteed, and only researchers could access the data. Information about the parent’s first name and home floor number was used for data matching. No incentive was given to the participants. The study was conducted in accordance with the Strengthening the Reporting of Observational Studies in Epidemiology (STROBE) reporting guideline for cohort studies.

### Measures

#### Internet Gaming Disorder

IGD symptoms were measured using the 9-item DSM-5 IGD symptoms checklist. It is a short, user-friendly, self-report measure assessing IGD symptoms of preoccupation, tolerance, withdrawal, unsuccessful attempts to limit gaming, deception or lies about gaming, loss of interest in other activities, use despite knowledge of harm, use for escape or relief of negative mood, and harm based on DSM-5 criteria [1]. Response options include no (0) and yes (1). A higher sum score indicates a higher level of IGD symptoms. A score of 5 is taken as the cutoff point for defining probable IGD. The Chinese version has been used with Chinese adults and children, which showed good psychometric characteristics [31,32]. The scale reliability was good in the present study (Cronbach alphas of 0.76 at T1 and 0.83 at T2).

#### Parental Psychological Control

The perception of parental psychological control of adolescents was measured using the Chinese version of the 8-item Psychological Control Scale-Youth Self-Report [33], which has been used in previous studies among Chinese adolescents and youth [11,21]. These items evaluate 3 psychologically controlling tactics (ie, guilt induction, invalidation of feelings, and love withdrawal). Sample item includes “My mother/father is always trying to change how I feel or think about things.” Participants responded on a 4-point Likert-type scale ranging from 1 (never) to 4 (always), with higher scores reflecting greater perception of parental psychological control. The Cronbach alphas of this scale were 0.79 at T1 and 0.83 at T2.

#### Parental Abuse

We used 2 items to measure the frequency of verbal and physical abuse by parents in the past year (ie, “How often have you been scolded or criticized by your parents” and “How often have you been physically punished by your parents”) [34]. The measure has been used with adolescents in previous studies [11,34]. Its responses range from 0 (never) to 4 (always), with higher scores indicating more frequent parental abuse. The Cronbach alphas were 0.69 and 0.67, and the correlation coefficients between the 2 items were 0.50 and 0.53 (large effect size), respectively, at the 2 time points.

#### Parental Support

We used 4 items to assess the level of perceived emotional and instrumental support from parents. Sample items include “How much support had you received from your parents when you needed to talk with someone or needed emotional support” and “How much support had you received from your family when you needed instrumental support (eg, financial support).” Responses were rated on a 7-point Likert scale ranging from 1 (none) to 7 (tremendous). Higher scores denote higher levels of perceived parental support. These items have been used in previous studies among Chinese adolescents and adults [11,35]. The Cronbach alphas of this scale in this study were 0.78 at T1 and 0.87 at T2.

#### Parent-Child Relationship

The quality of the relationship between parents and children was measured using a single item (ie, “How would you rate your relationship with your parents”). The item was rated on a 10-point scale, ranging from 1 (very poor) to 10 (very good). Higher scores denote better parent-child relationships. A previous study used this measurement among Chinese adolescents [11].

#### Background Factors

Background information, including sex; age; whether the participant was born in Kaifeng, Henan province; living arrangement; family socioeconomic status; and parental education levels, was collected.

### Statistical Analysis

SPSS 23.0 for Windows (IBM Corp, Armonk, NY) and Amos 24 were used for all statistical analyses. Descriptive statistics and correlation analyses of parental variables and IGD are presented. Linear regression models were performed to test the associations between background variables and IGD. The standardized coefficients (*β*), unstandardized coefficients (B), and corresponding 95% CIs are reported. To explore the reciprocal relationships between parental factors and IGD, a cross-lagged model was performed for each of the parental factors using the maximum likelihood method. Background variables that were significantly associated with IGD were controlled as covariates (ie, gender, single-parent family, educational level of mother). The goodness-of-model fit was assessed using the *χ*^2^/df value, comparative fit index (CFI), Bollen incremental fit index (IFI), Tucker-Lews index (TLI), root mean square error of approximation (RMSEA), and standardized root mean square residual (SRMR). For each index, the following criteria were applied to indicate a relatively good fit between the hypothesized model and the observed data: (1) *χ*^2^/df ratio ≤3; (2) CFI, IFI, TLI ≥0.90; (3) RMSEA, SRMR ≤0.08 [36-38]. *P* values less than .05 were considered statistically significant.

### Ethical Considerations

The study procedures were carried out in accordance with the Declaration of Helsinki. The study was approved by the Survey and Behavioral Research Ethics Committee of the corresponding author’s affiliation (reference #055-18). Written informed consent was obtained from the principals of all participating schools and all parents prior to data collection, and return of the completed questionnaire implied informed consent from participants.

## Results

### Descriptive Characteristics

The mean age of the participants was 15.6 years at baseline (T1), and about one-half of the participants (633/1200, 52.8%) were male. The majority of the participants (1035/1200, 86.3% at T1) lived with both of their parents and were from urban areas, while 62.8% (754/1200) of the participants’ mothers and 65.0% (780/1200) of their fathers had obtained an educational level of college or above. More than one-half (729/1200, 60.8%) of the participants rated their family socioeconomic status as good/very good (Table 1).

Of all the participants, 12.4% (148/1200) and 11.7% (140/1200) were classified as having probable IGD at T1 and T2, respectively. Among those without IGD at T1, 7.4% (78/1052) were classified with IGD at T2 (incidence rate), while among those with IGD at T1, 58.1% (86/148) remitted to IGD at T2 (remission rate).

**Table 1:** Descriptive characteristics of the participants at baseline (T1) and follow-up (T2; n=1200).

Background variables		T1	T2
Age (years), mean (SD)		15.60 (0.59)	—^a^
Gender (male), n (%)		633 (52.8)	—
Born in the investigated city, n (%)		1018 (84.8)	—
**Living with, n (%)**			
	Father	35 (2.9)	43 (3.6)
	Mother	87 (7.3)	97 (8.1)
	Both	1035 (86.3)	1019 (84.9)
	Neither	43 (3.6)	41 (3.4)
Single-parent family (no)		1070 (89.2)	1055 (87.9)
**Family socioeconomic status, n (%)**			
	Very poor	4 (0.3)	16 (1.3)
	Poor	28 (2.3)	28 (2.4)
	Ordinary	360 (30.0)	430 (35.8)
	Good	620 (51.7)	557 (46.4)
	Very good	109 (9.1)	101 (8.4)
	Refuse to answer	79 (6.6)	68 (5.7)
**Education level (father), n (%)**			
	Primary school or below	25 (2.1)	—
	Junior high school	122 (10.2)	—
	Senior high school/vocational school	251 (20.9)	—
	College	195 (16.3)	—
	Undergraduate	434 (36.2)	—
	Postgraduate	151 (12.6)	—
	Not applicable	22 (1.8)	—
**Education level (mother), n (%)**			
	Primary school or below	28 (2.3)	—
	Junior high school	140 (11.7)	—
	Senior high school/vocational school	251 (20.9)	—
	College	211 (17.6)	—
	Undergraduate	434 (36.2)	—
	Postgraduate	109 (9.1)	—
	Not applicable	27 (2.3)	—
IGD^b^ (yes), n (%)		148 (12.4)	140 (11.7)
IGD score^c^, mean (SD)		1.77 (2.09)	1.65 (2.27)
Parental psychological control^d^, mean (SD)		2.40 (0.64)	2.50 (0.67)
Parental support^e^, mean (SD)		5.20 (1.24)	5.26 (1.35)
Positive relationship with parents^f^, mean (SD)		8.22 (1.94)	7.93 (2.00)
Verbal or physical abuse by parents^g^, mean (SD)		1.19 (0.90)	1.19 (0.88)

^a^Not assessed.

^b^IGD: internet gaming disorder.

^c^Score ranges from 1 to 9.

^d^Score ranges from 1 to 4.

^e^Score ranges from 1 to 7.

^f^Score ranges from 1 to 10.

^g^Score ranges from 0 to 4.

### Associations Between Background Variables and Baseline IGD

Table 2 presents the associations between background factors and IGD. Female gender (*β*=–.14, *P*<.001) and higher maternal educational level (senior high school to college: *β*=–.23, *P*<.001; undergraduate or above: *β*=–.23, *P*<.001; reference group: junior high school or below) were negatively associated with IGD at T1. In addition, participants who lived in a single-parent family reported higher levels of IGD (*β*=.07; *P*=.04).

**Table 2:** Associations between background variables and internet gaming disorder (IGD) at baseline (T1; model statistics: R^2^=.065, *F*_12_=5.89, *P*<.001).

Background variables		Unstandardized coefficients		Standardized coefficient, *β*
		B (95% CI)	*P* value	
Gender (male vs female)		–.59 (–.84 to –.33)	<.001	–.14
Age (years)		–.07 (–.28 to .14)	.51	–.02
Living with both parents (yes vs no)		.19 (–.01 to .40)	.07	.06
Single family (yes vs no)		.46 (.02 to .91)	.04	.07
Family socioeconomic status		.03 (–.06 to .12)	.56	.02
**Educational level (father)**				
	Junior high school or below (reference)	N/A^a^	N/A	N/A
	Senior high school to college	.05 (–.42 to .51)	.84	.01
	Undergraduate or above	–.17 (–.68 to .34)	.51	–.04
	Not applicable	–.42 (–1.58 to .73)	.47	–.03
**Educational level (mother)**				
	Junior high school or below (reference)	N/A	N/A	N/A
	Senior high school to college	–1.00 (–1.44 to –.56)	<.001	–.23
	Undergraduate or above	–.96 (–1.45 to –.48)	<.001	–.23
	Not applicable	–.97 (–2.05 to .11)	.08	–.07

^a^N/A: not applicable.

### Correlations Between Parental Variables and IGD

As shown in Table 3, all parental factors including parental psychological control, parental support, a positive relationship with parents, and parental abuse at T1 were significantly correlated with IGD across the 2 waves (range for the absolute value of *r*: 0.16-0.29; all *P*<.01). Meanwhile, IGD at T1 was significantly correlated with all 4 parental variables at T1 and T2 (range for the absolute value of *r*: 0.15-0.29; all *P*<.01).

**Table 3:** Correlations between parental factors and internet gaming disorder (IGD).

Variable		Parental psychological control (T1^a^)	Parental psychological control (T2^b^)	Parental support (T1)	Parental support (T2)	Positive relationship with parents (T1)	Positive relationship with parents (T2)	Parental abuse (T1)	Parental abuse (T2)	IGD (T1)	IGD (T2)
**Parental psychological control (T1)**											
	*r*	1	0.54	–0.48	–0.31	–0.52	–0.33	0.56	0.36	0.29	0.18
	*P* value	—^c^	<.001	<.001	<.001	<.001	<.001	<.001	<.001	<.001	<.001
**Parental psychological control (T2)**											
	*r*	0.54	1	–0.33	–0.40	–0.38	–0.48	0.40	0.52	0.14	0.21
	*P* value	<.001	—	<.001	<.001	<.001	<.001	<.001	<.001	<.001	<.001
**Parental support (T1)**											
	*r*	–0.48	–0.33	1	0.51	0.65	0.38	–0.45	–0.29	–0.25	–0.17
	*P* value	<.001	<.001	—	<.001	<.001	<.001	<.001	<.001	<.001	<.001
**Parental support (T2)**											
	*r*	–0.31	–0.40	0.51	1	0.42	0.61	–0.35	–0.39	–0.15	–0.21
	*P* value	<.001	<.001	<.001	—	<.001	<.001	<.001	<.001	<.001	<.001
**Positive relationship with parents (T1)**											
	*r*	–0.52	–0.38	0.65	0.42	1	0.49	–0.54	–0.35	–0.28	–0.18
	*P* value	<.001	<.001	<.001	<.001	—	<.001	<.001	<.001	<.001	<.001
**Positive relationship with parents (T2)**											
	*r*	–0.33	–0.48	0.38	0.61	0.49	1	–0.33	–0.46	–0.15	–0.26
	*P* value	<.001	<.001	<.001	<.001	<.001	—	<.001	<.001	<.001	<.001
**Parental abuse (T1)**											
	*r*	0.56	0.40	–0.45	–0.35	–0.54	–0.33	1	0.53	0.26	0.21
	*P* value	<.001	<.001	<.001	<.001	<.001	<.001	—	<.001	<.001	<.001
**Parental abuse (T2)**											
	*r*	0.36	0.52	–0.29	–0.39	–0.35	–0.46	0.53	1	0.12	0.23
	*P* value	<.001	<.001	<.001	<.001	<.001	<.001	<.001	—	<.001	<.001
**IGD (T1)**											
	*r*	0.29	0.14	–0.25	–0.15	–0.28	–0.15	0.26	0.12	1	0.49
	*P* value	<.001	<.001	<.001	<.001	<.001	<.001	<.001	<.001	—	<.001
**IGD (T2)**											
	*r*	0.18	0.21	–0.17	–0.21	–0.18	–0.26	0.21	0.23	0.49	1
	*P* value	<.001	<.001	<.001	<.001	<.001	<.001	<.001	<.001	<.001	—

^a^T1: baseline.

^b^T2: follow-up.

^c^Not applicable.

### Cross-Lagged Models of Parental Variables and IGD at T1 and T2

The 4 cross-lagged models for parental factors and IGD showed good model fit (Table 4).

**Figure 1:**
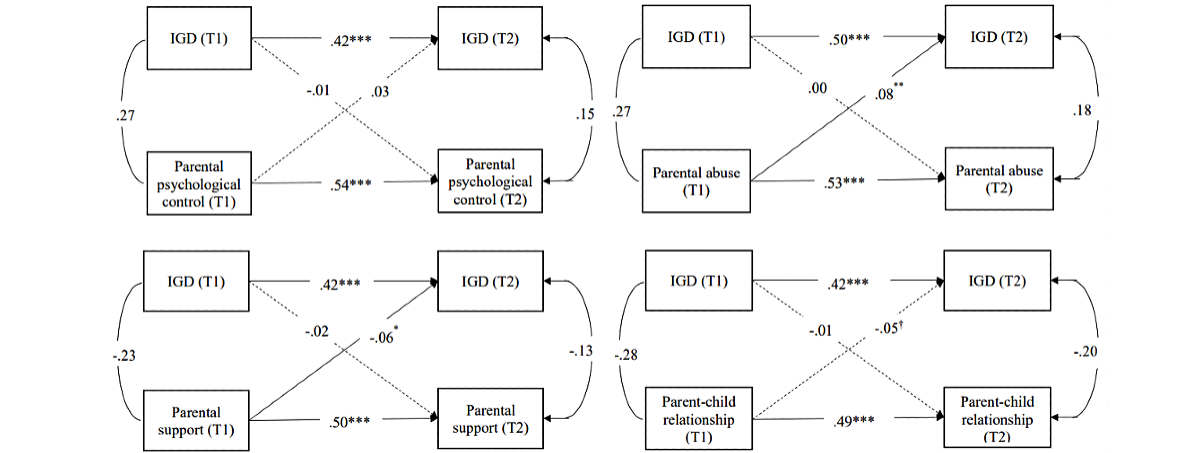
Cross-lagged models of parental variables and adolescent internet gaming disorder (IGD), showing the standardized coefficients and excluding the demographic variables that were controlled in the model (ie, gender, single family, and mother's education level. *P<.05, **P<.01, ***P<.001, †.05<*P*<.1; T1: baseline; T2: follow-up.

The path coefficients are shown in Figure 1. Parental support (*β*=–.06, *P*=.02) at T1 significantly predicted fewer IGD symptoms at T2, and parental abuse observed at T1 (*β*=.08, *P*=.002) predicted a higher level of IGD symptoms at T2, whereas parental psychological control (*β*=.03, *P*=.25) and parental relationship (*β*=–.05, *P*=.07) at T1 had nonsignificant effects on IGD symptoms at T2. In addition, all the paths from IGD symptoms at T1 to parental variables at T2 were not significant.

**Table 4:** Model fit indices of the 4 cross-lagged models for parental factors and internet gaming disorder (IGD).

Model	CMIN^a^(*χ*^2^)	*df*	CFI^b^	IFI^c^	RMSEA^d^	CMIN/*df*	SRMR^e^
Model 1 (Parental psychological control)	62.208	6	.937	.938	.088	10.368	0.06
Model 2 (Parental support)	79.788	7	.913	.914	.093	11.398	0.06
Model 3 (Parent-child relationship)	69.674	8	.927	.927	.080	8.709	0.06
Model 4 (Parental abuse)	64.616	9	.945	.946	.072	7.180	0.05

^a^CMIN: minimum value of the discrepancy function.

^b^CFI: comparative fit index.

^c^IFI: Bollen incremental fit index.

^d^RMSEA: root mean square error of approximation.

^e^SRMR: standardized root mean square residual.

## Discussion

### Principal Findings

This study represents the first longitudinal study to test the reciprocal relationships between various parental variables (ie, parental psychological control, parental support, positive relationship with parents, and parental abuse) and adolescent IGD in a large sample of Chinese first-year high school students. The results suggest that there were stable and significant cross-sectional correlations between parental factors and IGD symptoms. Furthermore, the cross-lagged effects indicate that higher levels of parental abuse positively predicted subsequent adolescent IGD symptoms, while parental support negatively predicted IGD symptoms. In contrast, there was no significant effect of IGD symptoms on predicting parental factors. The findings underline the need to tackle parental factors to prevent and reduce the risk of adolescent IGD.

We found that about 12% of the participants had probable IGD at both waves, which is comparable to the prevalence reported in previous studies among Chinese adolescents using the same measurement tool [11]. Only 52% of adolescents with probable IGD at T1 continued to have IGD at T2, suggesting that IGD symptoms may fluctuate across time. Similarly, previous studies reported low temporal stability of IGD, ranging from 20% to 50% across a 1-year period among adolescents [39]. This fluctuation may be explained by the rapid changes in biological, mental, and social status during adolescence. Such changes at this stage of life may be more frequent and have a more profound impact on one’s behaviors than in other stages (eg, adulthood) [29]. In addition, higher-grade high school students may perceive more academic stress as they prepare for the National College Entrance Examination and receive more stringent supervision from their teachers and parents, resulting in less time spent on the internet and lower levels of IGD symptoms [4,40,41]. Given that longitudinal studies on IGD are relatively lacking, the stability of IGD as a disorder is still under-examined. Surveillance studies with a longer follow-up period and during the transition stage are warranted to better understand the nature and conversion rate of IGD. Consistent with prior literature, adolescents who were male and living in single-parent families reported higher levels of IGD [42,43]. In addition, higher maternal educational levels may imply a better nurturing and upbringing environment for children, which may reduce the risk of adolescent IGD. Investigation and intervention efforts targeting these disadvantaged or at-risk populations (eg, male adolescents, single-parent family, and low maternal educational level) in particular are warranted.

Interestingly, we identified 2 significant predictors of IGD (ie, parental support and parental abuse), corroborating prior cross-sectional and longitudinal studies [11,14-16]. Parents are often primary caregivers of adolescents and provide emotional and instrumental support. Parental support is an important resource for resilience and ability to cope with stress by adolescents; such resources can reduce their stress, facilitate adaptive coping, and prevent risk behaviors [11]. Sufficient parental support may imply a positive family environment and family functioning, which have been negatively linked to various internalized (eg, depression and suicide) and externalized (eg, risky behaviors) adolescent problems [22]. Therefore, adolescents with sufficient parental support might be less likely to rely on the virtual world (ie, the internet) to meet their basic and psychological needs (eg, sense of belonging and connection) [22]. However, adolescents who perceive low parental support might feel rejected, feel incompetent, and develop a negative evaluation of oneself that leads to low self-esteem, which in turn is associated with a higher risk of internet addiction [44]. Future studies may explore other parental and familial factors (eg, family environment and functioning) for IGD and potential mediators (eg, needs satisfaction). Notably, 24% and 6% of the participants reported that they often or always experienced parental verbal and physical abuse, respectively. Such experiences could be extremely stressful and even traumatic for adolescents, and thus, they may overly rely on internet gaming to cope with stress and escape. This may support the application of the stress coping theory to understand the development of adolescent IGD. To our best knowledge, this is the first longitudinal study that showed parental abuse may increase adolescent IGD. However, it should be noted that the effect size for the associations between parental factors and IGD was relatively small. Interpretation of the findings should be cautious, and further longitudinal studies to confirm these findings are greatly warranted.

In contrast, parental psychological control did not predict subsequent IGD symptoms. This finding is inconsistent with that of a previous longitudinal study that indicated that parental psychological control, as an overly intrusive and authoritarian parenting style, had a significant effect on adolescent IGD [18]. However, adolescents with higher levels of parental psychological control may also perceive higher pressure of behavioral control from parents and inhibition of deviant behaviors such as internet gaming [45]; thus, parental psychological control may decrease adolescent IGD. The opposite effects of parental control on IGD may result in a nonsignificant relationship between parental psychological control and IGD. However, this speculation needs further investigation by including both parental psychological control and behavioral control. In addition, we found that a positive parental relationship predicted lower IGD symptoms at T2, with marginal significance (*P*=.07). A previous study suggested that the mother-child relationship was a stronger predictor of adolescent internet addiction than the father-child relationship [45]. Since mothers and fathers may have different weights and roles in the parent-child relationship, the fact that we combined mother and father as a parental variable may be a limitation of this study. Therefore, further studies should differentiate mother-child and father-child relationships, which might provide a better understanding of the role of the parent-child relationship on IGD.

However, the findings suggest that adolescent IGD did not significantly predict perceived parental status. Similarly, a previous study in the Netherlands reported that parental communication, reaction, and rules regarding internet use helped prevent children’s compulsive internet use, whereas compulsive internet use did not predict changes in parental communication and reactions toward children’s internet use [46]. The nonsignificant findings may not ascertain that adolescent IGD is not important in the family environment and parental-child relationship; a few emerging studies have suggested that excessive gaming may displace opportunities for family interaction [22]. It is plausible that the status of the family environment and parent-child relationship are relatively stable attributes, and a longer follow-up period may be required to observe potential changes in parental status. In addition, the effect of IGD on parental responses may be based on the prerequisite that parents are aware of their children’s gaming behavior and consider it a problem. In other words, these factors may moderate the effect of adolescent IGD on parental responses, which should be assessed as confounders in future studies.

This study has several limitations. First, participants were selected based on convenience sampling from central China, which could be subject to selection bias and reduce the generalizability of findings to adolescents from other regions. Second, our results had relatively small effect sizes. Given the large sample size, associations with small effect sizes could be statistically significant (eg, weak prospective associations between parental factors and IGD); both statistical significance and effect sizes should be taken into consideration when interpreting the results. Third, the use of self-reported measures might be subject to recall bias and social desirability bias. Disclosing IGD status may be a sensitive issue for students during school-based surveys, even when anonymity is guaranteed. In addition, the variables of parent-child relationship, physical abuse, and verbal abuse were measured by single-item scales. Future studies should validate the findings with more refined measures. Future research may also consider incorporating data from multiple informants (eg, parents) and different methods (eg, qualitative interviews). Meanwhile, the Cronbach alphas for the parental abuse scale were relatively low (<0.7), which might be attributed to the limited number of items in the scale [47-49]. Fourth, as we focused on parental factors, other individual-level (eg, emotional regulation and coping) and interpersonal-level factors (eg, peers and teachers) of adolescent IGD were not investigated. Future studies may investigate the interplay of multilevel factors in the development of IGD.

Nevertheless, the findings of this study signify the need for parents to be involved in interventions for adolescents exhibiting symptoms of IGD to increase the effectiveness of treatment. Education and skill training programs for parents could increase their awareness of the potential harms of their psychological, physical, and verbal abuse on IGD for adolescents and teach them proper parenting practice (eg, positive parenting styles) and intergenerational communication (eg, nonviolent communication skills). In addition to individual-based therapy such as cognitive-behavioral and problem-solving therapy, family-based therapy that views children’s problems from a familial or ecological perspective and helps to foster a supportive and healthy family environment may be particularly helpful. Indeed, the family-based approach has shown efficacy in reducing the risk of IGD. For instance, multi-family group therapy, which involves reducing high and unreasonable expectations and criticism by parents and identifying alternative ways for adolescents to satisfy needs for competence and relatedness, has effectively reduced the rate of gaming problems among Chinese adolescents [50].

### Conclusions

This study indicates that parental support and parental abuse were salient predictors of adolescent IGD. However, the longitudinal effect of IGD on parental factors was nonsignificant. These findings enhance the understanding of the prospective associations between parental factors and adolescent IGD in the Chinese context and demonstrate the value of fostering positive family dynamics and environment in adolescent IGD interventions.

## Abbreviations

CFI: comparative fit index
DSM-5: Diagnostic and Statistical Manual, Fifth Edition
ICD: International Classification of Diseases and Related Health Problems
IFI: incremental fit index
IGD: internet gaming disorder
RMSEA: root mean square error of approximation
SRMR: standardized root mean square residual
STROBE: Strengthening the Reporting of Observational Studies in Epidemiology
TLI: Tucker-Lews index
