# Correction: Parental Factors Associated With Internet Gaming Disorder Among First-Year High School Students: Longitudinal Study

**DOI:** 10.2196/44458

**Published:** 2022-12-06

**Authors:** Rui She, Youmin Zhang, Xue Yang

**Affiliations:** 1 Department of Rehabilitation Sciences The Hong Kong Polytechnic University Hong Kong China; 2 The Jockey Club School of Public Health and Primary Care Faculty of Medicine The Chinese University of Hong Kong Hong Kong China

In “Parental Factors Associated With Internet Gaming Disorder Among First-Year High School Students: Longitudinal Study” (JMIR Serious Games 2022;10(4): e33806) the authors noted one error.

In the originally published article, the equal contribution note indicating that Rui She and Youmin Zhang contributed equally to the manuscript, was missed and was published as follows:

Rui She^1,2^, PhD; Youmin Zhang^1^, MPH; Xue Yang^1^, MPhil, PhD

^1^The Jockey Club School of Public Health and Primary Care, Faculty of Medicine, The Chinese University of Hong Kong, Hong Kong, China

^2^Department of Rehabilitation Sciences, The Hong Kong Polytechnic University, Hong Kong, China

The author metadata has been corrected to include the 'equal contribution' footnote:

Rui She^1,2*^, PhD; Youmin Zhang^1*^, MPH; Xue Yang^1^, MPhil, PhD

^1^The Jockey Club School of Public Health and Primary Care, Faculty of Medicine, The Chinese University of Hong Kong, Hong Kong, China

^2^Department of Rehabilitation Sciences, The Hong Kong Polytechnic University, Hong Kong, China

^*^these authors contributed equally

The correction will appear in the online version of the paper on the JMIR Publications website on December 06, 2022, together with the publication of this correction notice. Because this was made after submission to PubMed, PubMed Central, and other full-text repositories, the corrected article has also been resubmitted to those repositories.

